# To Prevent Rotation in Fracture of the Femur, Etc.

**Published:** 1874-08

**Authors:** J. E. O’Brien

**Affiliations:** Scranton, Pa.


A MONTHLY RECORD OF

*Medicine, Surgery and the Collateral Sciences.*

EDITED BY J. ADAMS ALLEN, M.D., LL.D.; AND WALTER HAY, M.D.

VOL. XXXI.—AUGUST, 1874.—NO. 8.

### Original Communications.

ARTICLE I.—*To Prevent Rotation in Fracture of the Femur, when treated by Weight and Pulley.* By J. E. O'BRIEN, M.D., Scranton, Pa.

As extension, and sometimes counter-extension, can be best maintained by adhesive plaster, in the early stage of fracture of the femur, it only remains to apply the same means to prevent rotation. If I treat the right limb I wind two straps around it at the knee, and tack them to the sides of the bed; the right strap leaves the popliteal surface, the left strap leaves the patella. I wind one of them just above the patella, the other just below. This is the best plan yet tried to prevent rotation; I have used it in two cases.

While writing about this fracture, I will mention an easy method of improvising a good thigh splint, which I think will be appreciated by my fellow students in the country. Having put on the weight for extension, take binders' board or leather, measure from the patella to the pubes, and round the thigh, above and below, cut accordingly; soften the splint in hot water, slip it beneath the thigh, tuck in an even layer of cotton on each side till the cotton meets behind; pass four leather straps with buckles under your splint, mould it to the limb, using more cotton if necessary, and draw the straps tight. This is an efficient splint, but I have suited myself still better by having it cut into strips after it has been on a few days, and uniting them hinge fashion, by strips of adhesive plaster.

With such means for preventing rotation, such facility in improvising short thigh splints, and with the sand bags to support the leg, I consider the weight and pulley treatment almost perfect, and have used it in the Lackawanna Hospital here, and in private practice. Just now there is a discussion going on in New York on the treatment of this fracture, and an effort is being made to introduce plaster of Paris as a *primary* dressing.

